# Hybrid Quantum Dot Light-Emitting Diodes for White Emission Using Blue Phosphorescent Organic Molecules and Red Quantum Dots

**DOI:** 10.3390/mi10090609

**Published:** 2019-09-14

**Authors:** Aram Moon, Jiwan Kim

**Affiliations:** Department of Advanced Materials Engineering, Kyonggi University, Suwon 16227, Korea; moonar05200@gmail.com

**Keywords:** quantum dots, phosphorescent molecules, white emission

## Abstract

Hybrid quantum dot light-emitting diodes (QLEDs) with no buffer layer were developed to achieve white emission using red quantum dots by spin-coating, and blue phosphorescent organic molecules by thermal evaporation. These unique bichromatic devices exhibit two distinct electroluminescent peaks with similar intensities at 10.5 V. For white emission, these hybrid QLEDs present a maximum luminance of 6195 cd/m^2^ and a current efficiency of 2.02 cd/A. These results indicate that the unique double emission layers have the potential for bright and efficient white devices using fewer materials.

## 1. Introduction

Colloidal quantum dots (QDs) have an excellent photoluminescence quantum yield (up to 97%) [1,2] and their color can be tuned by controlling their size. Recently, electrically driven quantum dot light-emitting diodes (QLEDs) have become the next-generation display platform owing to their superior optical properties and convenient solution processability. Since the first QLEDs were reported in 1994 [3], many groups have studied the development of balanced charge transport layers as well as efficient QDs [4,5,6,7]. In particular, the adaptation of ZnO-based inorganic electron transport layers (ETLs) has dramatically improved the brightness and the device efficiency of QLEDs [8,9,10].

For practical full-color display applications, white emitting devices should be the next step over monochromatic devices. However, only limited white electroluminescence (EL) devices using QDs have been reported, for two main reasons. First, the emission layer (EML) using red, green, and blue mixed QDs showed inevitable energy loss to adjacent QDs [11]. Second, the stacked tandem structure is another way to achieve white emission, but its complicated fabrication cannot be avoided because of in-between buffer layers as well as multiple EMLs [12]. Our group reported highly efficient white QLEDs with blue and green mixed QDs and red organic phosphorescent molecules, which had a maximum luminance of 20,453 cd/m^2^ and an external quantum efficiency (EQE) of 9.19% [13]. However, there are other ways to reduce the number of emitting materials for white emission. Intriguingly, white light-emitting diodes by controlling the thickness of silicon nanocrystals and organic dye have been reported [14].

In this study, we fabricate white standard structured QLEDs with double EMLs using red QDs and blue organic phosphorescent molecules. After the red EML (rEML) is formed with only red QDs by spin-coating, the blue EML (bEML) is deposited by the thermal evaporation of blue organic phosphorescent molecules on rEML. Recently, a structure of QD color filters with organic light-emitting diodes (OLEDs) used by major display manufacturers, which mainly applies QDs to the color converting layer [15,16], has been attracting a great deal of attention from the display industry. This QD-OLED structure is different from our white QLEDs, but there is a common use of blue OLEDs in both structures. In this study, we use bis[2-(4,6-difluorophenyl)pyridinato-C2,N]iridium(III) (FIrpic) as blue emitting organic molecules. By using FIrpic, which has a broader emission spectrum than QDs, white emission with a high color rendering index (CRI) can be achieved with fewer materials, i.e., only two emitting materials. The device exhibits a maximum luminance of 6195 cd/m^2^, a current efficiency of 2.02 cd/A, and an EQE of 1.01%. For an applied voltage of 10.5 V, the Commission Internationale de l’Eclairage (CIE) coordinates are positioned to a white point of (0.35, 0.33) with a high CRI of 60. A shift of the color coordinates according to the applied voltage is observed owing to changes in the EL peaks.

## 2. Materials and Methods

The white QLEDs were fabricated on indium-tin-oxide- (ITO) coated glass substrates. The substrates were sequentially cleaned with isopropyl alcohol and then rinsed with deionized water. After the patterned ITO substrates were treated in ultraviolet-ozone for 15 minutes, poly(3,4-ethylenedioxythiophene):poly(styrene sulfonate) (PEDOT:PSS) was spin-coated onto the ITO substrate as the hole injection layer (HIL) at 3000 rpm for 35 seconds and dried on a hot plate for 30 minutes at 150 °C. For the hole transport layer (HTL), poly[(9,9-dioctylfluorene-co-N-[4-(3-methylpropyl)]-diphenylamine] (TFB) was dissolved in chlorobenzene (8 mg/mL). The resulting mixture was spin-coated onto the PEDOT:PSS layer at 2000 rpm for 35 seconds. The resulting assembly was then dried on a hot plate at 150 °C for 30 minutes. For the fabrication of hybrid double EMLs, CdSeS/ZnS QDs (In-visible Inc., Suwon, Korea) for the rEML were dissolved in hexane with a concentration of 10 mg/mL and then spin-coated on top of the TFB layer at 2000 rpm for 5 seconds. The organic molecules and metals were deposited in continuance by thermal evaporation without breaking vacuum. In addition, 1,3-Bis(carbazol-9-yl)benzene (mCP) + FIrpic for the bEML, 2′,2′,2″-(1,3,5-benzinetriyl)-tris(1-phenyl-1-H-benzimidazole) (TPBi) for the ETL, and LiF/Al for the cathode were thermally evaporated with a deposition rate of approximately 2 Å/s for mCP + FIrpic, 1 Å/s for TPBi, 0.5 Å/s for LiF, and 3 Å/s for Al electrode, respectively. The current density–voltage–luminance (J-V-L) characteristics of the devices were measured using a spectroradiometer (CS-2000, Konica Minolta Holdings, Inc., Tokyo, Japan) with a source meter (Keithley-2400, Keithley Co. Ltd., Beaverton, Oregon, USA) under ambient conditions. From these J-V-L measurements, changes in the luminance and current efficiency of the fabricated devices as a function of the applied voltage were systematically studied.

## 3. Results and Discussion

**Figure 1:**
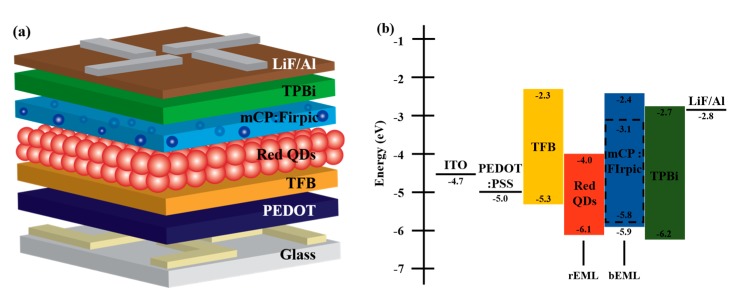
(**a**) Device structure and (**b**) energy band diagram of white standard structured quantum dot light-emitting diodes (QLEDs) with double emission layers (EMLs) [17].

Figure 1a illustrates white-emitting hybrid QLEDs with double EMLs comprising ITO/PEDOT:PSS (40 nm)/TFB (40 nm)/Red QDs (rEML, 10 nm)/mCP:FIrpic (bEML, 20, 30 nm)/TPBi(30, 40 nm)/LiF(1 nm)/Al (100 nm). In this study, unique double EMLs were designed for simultaneous red and blue emissions from the QDs and FIrpic, respectively. The organic molecules, mCP and FIrpic, were selected as the host and dopant materials and deposited directly onto the red QD layer as the bEML using an evaporation method with no buffer layer. As shown in Figure 1b, PEDOT:PSS was used as the HIL on an ITO anode to match with the anode work function. TFB was used as the HTL to transport holes efficiently from PEDOT:PSS to the highest occupied molecular orbital of the red QDs. Because of the chemical inertness of the TFB thin films to nonpolar solvents, red QDs in hexane were successfully spin-coated onto the TFB layer for the rEML with no chemical damage. The entire device features a sophisticated design for balanced charge transport from each electrode to central EMLs.

**Figure 2:**
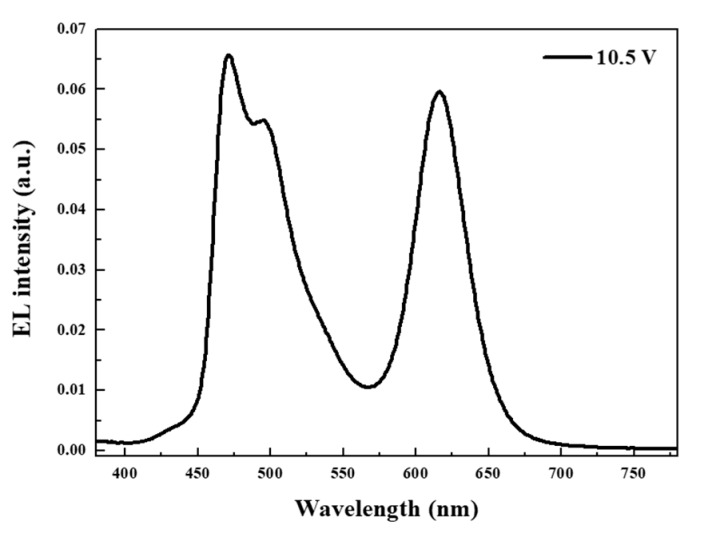
Electroluminescence (EL) spectrum of hybrid QLEDs with double EMLs for an applied voltage of 10.5 V.

**Figure 3:**
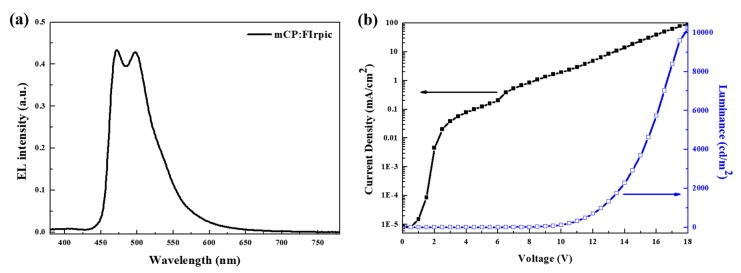
(**a**) EL spectrum and (**b**) current density–luminance with applied voltage of OLEDs with 1,3-Bis(carbazol-9-yl)benzene (mCP): bis[2-(4,6-difluorophenyl)pyridinato-C2,N]iridium(III) (FIrpic) as an EML.

Figure 2 presents the EL spectrum of the white-emitting hybrid QLEDs. During the fabrication process, the device was transferred to the thermal evaporator for the formation of bEML after spin-coating rEML. Contrary to the expected behavior that phosphorescent organic molecules are sensitive to the solution process, narrow red emission from QDs in the rEML and traditional blue emission from FIrpic in the bEML were simultaneously observed for an applied voltage of 10.5 V. In Figure 3, blue OLED devices (ITO/PEDOT:PSS/TFB/mCP:FIrpic/TPBi/LiF/Al) were separately fabricated to evaluate the feasibility of phosphorescent organic materials on the solution-processed TFB layer. Moreover, stable optical/electrical properties were demonstrated.

**Figure 4:**
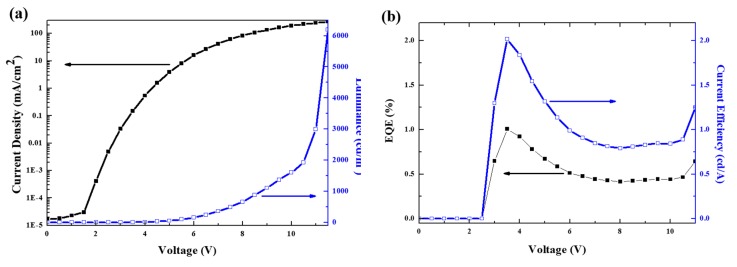
Characteristics in (**a**) current density–luminance with applied voltage and (**b**) external quantum efficiency (EQE)—current efficiency with applied voltage of hybrid QLEDs with double EMLs.

Figure 4a presents the voltage-dependent variations of the luminance and current density of the hybrid QLEDs with double EMLs. A peak luminance of 6195 cd/m^2^ was achieved at 11.5 V (corresponding to a current density of 269.58 mA/cm^2^). Owing to the thick double EMLs, the turn-on voltage was shifted to a higher voltage region than general monochromatic QLEDs. In Figure 4b, a maximum EQE of 1.01% and a current efficiency of 2.02 cd/A were obtained for a current density of 0.15 mA/cm^2^.

**Figure 5:**
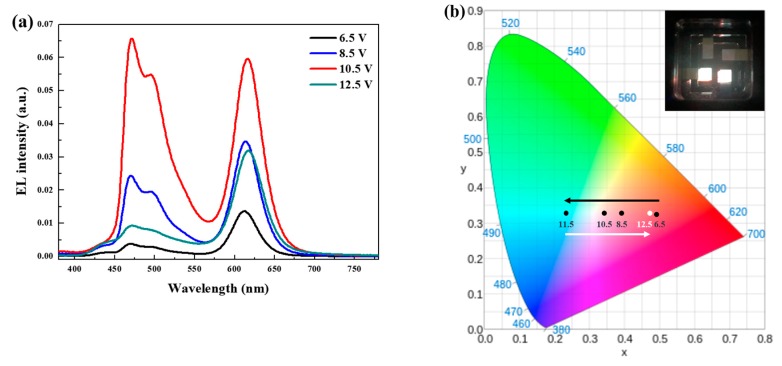
Voltage-dependent (**a**) EL spectra and (**b**) Commission Internationale de l’Eclairage (CIE) coordinates of hybrid QLEDs with double EMLs. The inset shows the white emission from 2.5 mm × 2.5 mm pixels at 10.5 V.

The spectral variations of the EL spectra and the shift of the CIE coordinates of the hybrid QLEDs with double EMLs at different voltages are shown in Figure 5a,b, respectively. Firstly, hybrid QLEDs emitted red light, then shifted to white as the voltage increased up to 10.5 V. As demonstrated in Figure 5a, this shift resulted from the later emissions of FIrpic in bEML at a higher voltage region. Therefore, the CIE coordinates shifted from (0.49, 0.32) at 6.5 V to (0.35, 0.33) at 10.5 V with a CRI of 60. Both blue and red emissions decreased after exhibiting balanced white emission at 10.5 V, but the decrement of blue emission was dominant at a higher applied voltage. Therefore, the CIE coordinates returned to red at 12.5 V. The hybrid QLEDs emitted white light at a lower applied voltage than our previous results [13] because QLEDs with red QDs showed a relatively lower turn-on voltage than those with blue QDs, owing to their small bandgap [8,18].

**Figure 6:**
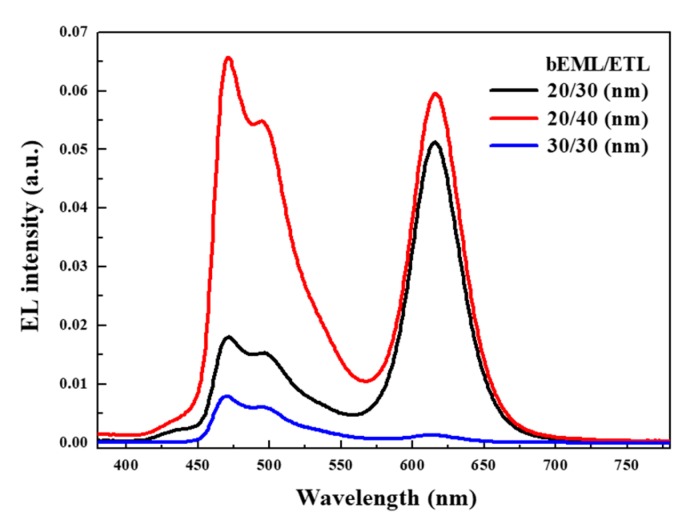
EL spectra of QLEDs with various thicknesses of blue emission layer (bEML) and electron transport layer (ETL) at 10.5 V.

Figure 6 shows the EL spectra of the hybrid QLEDs with various thicknesses of bEML and ETL at the same applied voltage of 10.5 V. In standard structured QLEDs, the transport of electrons from the cathode can be slowed down as ETL becomes thicker. Therefore, the strong blue emissions of the hybrid QLEDs with 40 nm ETL result from an upward shift of the recombination zone (RZ) to bEML. Interestingly, for hybrid QLEDs with 30 nm bEML and 30 nm ETL, the entire EL emission was very weak even though the combined thickness of bEML and ETL is the same as 60 nm. It is assumed that the thickness of bEML is more critical than that of ETL for the effective transport of electrons to the RZ, and it is also supported by the dramatic change of red emission from QDs in rEML with the same thickness of ETL. Consequently, hybrid QLEDs with 20 nm bEML and 40 nm ETL are ideal for achieving balanced white emission due to the adequate location of the RZ at the interface of rEML and bEML.

These hybrid QLEDs demonstrated two distinct emissions simultaneously with no buffer layer between rEML and bEML, which is typically required to protect the delicate organic molecules. More importantly, hybrid QLEDs have unique heterogeneous EMLs with inorganic QDs and organic mCP:FIrpic. As shown in Figure 2, the asymmetric shape of the EL peak from FIrpic helped to cover the green region in the entire visible spectrum. Therefore, the white emission with a high CRI of 60 achieved with hybrid QLEDs was mostly due to the broad blue emission from FIrpic. Unlike our previous report [13], fewer materials (only one red QD and one blue OLED) were used to accomplish white emission at a relatively low applied voltage and it provided a huge advantage in terms of reduced manufacturing costs and fabrication steps.

## 4. Conclusions

Hybrid white-emitting QLEDs with blue phosphorescent organic molecules and red QDs were successfully fabricated. The EL spectra of the device exhibited two emission peaks from each layer simultaneously. The hybrid QLEDs with 20 nm bEML and 40 nm ETL had a maximum luminance of 6195 cd/m^2^, an EQE of 1.01%, and a current efficiency of 2.02 cd/A, while emitting white light with a high CRI of 60 at 10.5 V. The thickness of bEML is more important than that of ETL for balanced white emission. On increasing the applied voltage, the emission arises from the red QDs firstly, followed by blue EL from FIrpic. Therefore, the CIE coordinates moved from the red to the white region according to the increment of the applied voltage.
